# Alzheimer’s Disease, Mild Cognitive Impairment and Mediterranean Diet. A Systematic Review and Dose-Response Meta-Analysis

**DOI:** 10.3390/jcm10204642

**Published:** 2021-10-10

**Authors:** Natalia García-Casares, Paloma Gallego Fuentes, Miguel Ángel Barbancho, Rosa López-Gigosos, Antonio García-Rodríguez, Mario Gutiérrez-Bedmar

**Affiliations:** 1 School of Medicine, University of Málaga, 29010 Málaga, Spain; paloma_gf_96@hotmail.com (P.G.F.); mabarbancho@uma.es (M.Á.B.); gigosos@uma.es (R.L.-G.); antoniogr@uma.es (A.G.-R.); 2 Centro de Investigaciones Médico-Sanitarias (CIMES), Campus de Teatinos, University of Málaga, 29010 Málaga, Spain; 3 Instituto de Investigación Biomédica de Málaga-IBIMA, 29010 Málaga, Spain; 4 CIBERCV Cardiovascular Diseases, Carlos III Health Institute, 28029 Madrid, Spain

**Keywords:** Mediterranean diet, Alzheimer’s disease, mild cognitive impairment, meta-analysis

## Abstract

Alzheimer’s Disease (AD) is a pathology with increasing prevalence in the context of a more long-lived society and it is the first cause of dementia in western countries. It is important to investigate factors that can be protective and may influence its development, in order to act on them trying to reduce AD incidence and its progression. The aim of this study was to conduct a systematic review and meta-analysis to determine the effects of a higher adherence to Mediterranean diet (MD) on Mild Cognitive Impairment (MCI) and AD. A literature search in PubMed, The Cochrane Library Plus and Scopus was conducted, selecting articles that analyzed associations between MD adherence and AD biomarkers (Volumetry assessed by MRI and betamiloide and Tau deposits by PET); cognitive performance in patients at risk or presenting MCI and AD; and incidence or progression from MCI to AD. Out of the 589 studies screened, 22 studies met eligibility criteria for the systematic review and qualitative synthesis. Finally, 11 studies were included in the meta-analysis (12,458 participants). Higher adherence to MD was associated with a significantly lower risk of MCI (RR = 0.91, 95%CI = 0.85–0.97) and lower risk of AD (RR = 0.89, 95% CI = 0.84–0.93). Our results enhance the importance of taking health-promoting lifestyle measures like following Mediterranean dietary patterns in order to reduce AD risk.

## 1. Introduction

Alzheimer’s disease (AD) is the predominant type of dementia in the world, accounting for 60 to 70% of all cases [1,2]. Due to the improvement of living standards and the resulting increase of life expectancy, AD worldwide prevalence is expected to triple by 2050 [1,3], leading this rapidly increase to many social and economic costs [4].

World Health Organization recognizes dementia as a public health priority. In May 2017, the World Health Assembly developed the “Global action plan on the public health response to dementia 2017–2025 aims” for enhance life quality of dementia patients and their caregivers, as well as reduce the impact of that disease in communities and countries. Among its risk reduction targets, adoption of healthy dietary patterns is included [2]. The plan also emphasizes the need to conduct multi-sectorial evidence-based interventions affordable for most people to encourage them to make proactively healthy lifestyle changes and reduce exposure to modifiable risk factors, so that the rapidly increase of this disease could be slowed [4].

Currently, no preventive or curative treatment for AD is available. Cholinesterase inhibitors and N-methyl-D-aspartate receptor antagonists can be used to reduce some cognitive symptoms and are the only farms approved by the Food and Drug Administration as AD medications, while antipsychotic and antidepressant drugs can help control behavioral and psychological symptoms. Nevertheless, disease-modifying treatments are still under research [5], and for that reason, giving scientific evidence in possible risk and protective factors for AD assume high significance.

Age (which is the strongest known AD risk factor [4]), being female and carrying APO-E4 gene allele are non-modifiable AD risk factors [6], which interact with environmental and biological factors, modifying AD risk. It is noted that one of every three new AD cases is due to risk factors that are modifiable [1,3,7]. Some of these modifiable risk factors with established scientific evidence are physiological risk factors (mid-life hypertension, diabetes, obesity or inflammation), concomitant diseases (peripheral arterial disease, low cardiac output or depression) and lifestyle factors (education, smoking, physical activity or diet) [6]. 

Related to diet, Mediterranean Diet (MD) is typified by high consume of vegetables, fruits, legumes, nuts and whole grains, olive oil as the main fat source, moderate consume of fish, low to moderate consume of dairy products, low consume of poultry, meat and saturated fatty acids, and moderate consume of alcohol only in meals [8,9,10,11,12]. 

Numerous studies consistently support a role of MD in the primary and secondary prevention of cardiovascular disease (CVD), by improving cardiometabolic health and glycaemic control [13,14]. Additionally, scientific evidence demonstrates MD effects on cognition. A recent meta-analysis concluded that high adherence to MD reduced the risk of global cognitive decline in non-demented older adults over 60 years of age [15]. Adherence to MD is included in a lifestyle pattern influenced by sociocultural, educational, family and economic factors [16,17]. For that reason, the protective effect of MD in AD could be explained, rather than by a single mechanism, by a complex set of pathways in which dietary components and other lifestyle factors take part, interacting synergistically and additively with each other. 

These interactions could contribute directly to reduce AD risk (by its neuroprotective effects) as well as indirectly (being protective factors of cardiovascular and metabolic diseases, which are themselves risk factors for AD) [3,8,9,14]. In this line, Previous reviews and meta-analysis showed that MD was associated with improved cognitive function, a decreased risk of mild cognitive impairment (MCI) or decreased risk of dementia and AD [18,19]. Furthermore, changes in AD biomarkers (such as β-amyloid (βA) deposition, Tau phosphorylation, cortical thickness or glucose metabolism in brain) precede clinical AD symptoms by 10–20 years [1,8]. Accordingly, the adoption of preventive lifestyle measures should be applied from as early in life as possible. In fact, implementing preventive measures that reduce AD incidence or delay its progression could potentially minimize AD prevalence by nearly 9 million cases during the next 40 years [20]. A meta-analysis studied the relationship of diet with respect to the hallmark AD biomarkers (tau and beta-amyloid) and found that most of the MD studies, showed that adherence to the MD reduces significantly AD biomarker burden [21].

Previous systematic reviews and meta-analyses have concluded that a higher adherence to MD was inversely associated with cognitive decline. However, they have either analyzed a small number of studies [12], or they were focused on MCI and dementia in general including other non-AD dementia [18]. Therefore, the aim of this systematic review is to analyze, from a clinical point of view, the latest and most complete scientific evidence to support MD adherence as a protective factor for AD, and its impact on cognition. In order to focus exclusively on the effects of the adherence to MD on cognition, and the incidence or progression to MCI or AD (excluding other types of dementia or diet), we conducted an updated systematic review and dose-response meta-analysis of all studies examining the impact of MD on cognition in people at risk for AD, presenting MCI or AD patients.

## 2. Materials and Methods

To conduct this systematic review and meta-analysis, we followed the PRISMA (Preferred Reporting Items for Systematic Reviews and Meta-Analyses) statement guidelines checking each of its 27 items in each phase of the process [22].

### 2.1. Eligibility and Selection Criteria

The general eligibility criteria consisted of quantitative studies that investigated as a primary or secondary outcome the associations between adherence to MD with the occurrence of MCI and/or AD. To be eligible for inclusion in this systematic review and meta-analysis, the study design had to be either an observational study (cross-sectional, case-control or longitudinal cohort studies) or a randomized controlled trial. Case-series and reviews were not considered. All studies included in the meta-analysis also had to report adequate information to quantify MD adherence and a risk estimate (odds ratio [OR], hazard ratio [HR] or relative risk [RR]) for MCI and/or AD, or data from which it could be calculated.

Articles were included if they studied (1) associations between adherence to MD and AD preclinical biomarkers (βA plaques or Tau tangles deposition, glucose metabolism in brain or brain volumes measured with neuroimaging techniques or cerebrospinal fluid) in non-AD participants but with AD risk; (2) cognitive performance in participants at risk of AD or who are already affected by this disease, measured through neuropsychological tests (NPT); and (3) incidence or longitudinal progression from MCI to AD (all of these outcomes obtained from humans). 

Studies were excluded if they (1) did not include MD; (2) analyzed effects of Modified Mediterranean Ketogenic Diet or individual components of MD; or (3) related other dementias subtypes not AD such vascular dementia, Lewy body and frontotemporal dementias without including outcomes in relation to AD. For quantitative analysis, articles where cognition was not measured objectively by neuropsychological tests, or the diagnosis was based on medical reports were excluded. We also excluded animal studies, letters to editor, editorials, book chapters, no original articles, reviews, trial protocols, and articles that either were not written in English or Spanish 

### 2.2. Search Strategy

A systematic literature search using PubMed, Scopus and The Cochrane Library Plus was performed until June of 2021. The search strategy was conducted using the Medical Subject Headings (MESH) terms “Mediterranean diet”, “Alzheimer Disease”, “Cognitive Dysfunction” and “Mild Cognitive Impairment” and Boolean operators AND/OR for PubMed, Scopus and The Cochrane Library Plus. No chronologic or other restrictions were introduced. In addition, we scrutinized references from relevant original papers and review articles to identify further pertinent studies. The complete search strategy is shown in Appendix A.

An initial screening was conducted for the exclusion of duplicate references and irrelevant articles. Three authors (NGC, PGF and MGB) independently reviewed all identified abstracts for eligibility. All abstract reporting on the association between MD and MCI or AD were selected for full text review.

### 2.3. Data Extraction

Two reviewers (NGC and MGB) independently extracted data form the included studies using a standardized predesigned form and any disagreement was resolved by mutual consensus in the presence of a third investigator (PGF). The following data were recorded form each study: first author’s name, year of publication, study location, follow-up duration, sample size, age, diet and study-related measures and the measure and strength of the association HR or OR with corresponding 95% confidence interval (CI).

### 2.4. Risk of Bias Assessment

The risk of bias was assessed using the Newcastle-Ottawa Quality Assessment Scale (NOS) for observational studies [23]. This scale examines potential bias on selection, comparability, and outcome. The total NOS score ranges from 0 to 10 for cross-sectional studies, and from 0 to 9 for case-control and cohort studies. We identified total scores ≤4 as high risk of bias, scores 5–6 as moderate risk of bias, and scores ≥7 indicated a low risk of bias.

### 2.5. Statistical Analysis

All types of associations were estimated as RRs and 95% CIs. HRs were directly considered as RRs and, where necessary, ORs were transformed into RRs with the use of the outcome incidence in the nonexposed group [24]. Because these transformations can underestimate the variance or the RRs derived from the ORs [25,26], we performed two sensitivity analyses, one that excluded three studies for which this transformation has been applied [8,16], and a second sensitivity analysis in which ORs were not transformed into RRs. One article which reports both outcomes MCI and AD [16] was treated as two separate studies. Adherence to MD was assessed according to a MD score ranging from 0 (lowest adherence) to 9 (highest adherence) in all studies except one [27] where a MD score ranging from 0 (lowest adherence) to 55 (highest adherence) was used. For this study, we converted the RR estimated using a 55-point scale to the corresponding RR in a 9-point scale. Results from multivariable models with the most-complete covariate adjustments were used. We computed a RR with 95% CI for an increase of one unit for each report. For those studies where adherence to MD were not introduced as continuous variable into the models, we used the method described by Greenland and Longnecker [28] and Orsini and colleagues [29] to calculate the trend from the correlated estimates for log RR across categories of MD adherence. The median or mean MD score in each category was used as the corresponding dose of adherence. The midpoint of lower and upper bounds was regarded as the dose of each category if the study only reported the range. Pooled results were reported separately for MCI, AD, and the composite end point of any of these outcomes. To visually assess the RR estimates and corresponding 95% CIs across studies, we generated a forest plot shorted by year of publication. A subgroup analysis according to study design was also performed.

To assess heterogeneity of relative risks across studies, we examined forest plots and used Cochran’s Q tests (with a significance level of *p* ≤ 0.10) and I^2^ statistics. I^2^ > 30% was considered at least moderate heterogeneity. In the presence of heterogeneity, random effects models (DerSimonian and Laird method) were used rather than fixed effects models (Mantel-Haenszel method). Potential publication bias was assessed through visual inspection of funnel plot and by Egger’s and Begg’s tests.

All statistical analyses were performed with Stata version 17.0 (Stata Corp). A *p* value <0.05 was considered statistically significant, except where otherwise specified. 

## 3. Results

**Figure 1:**
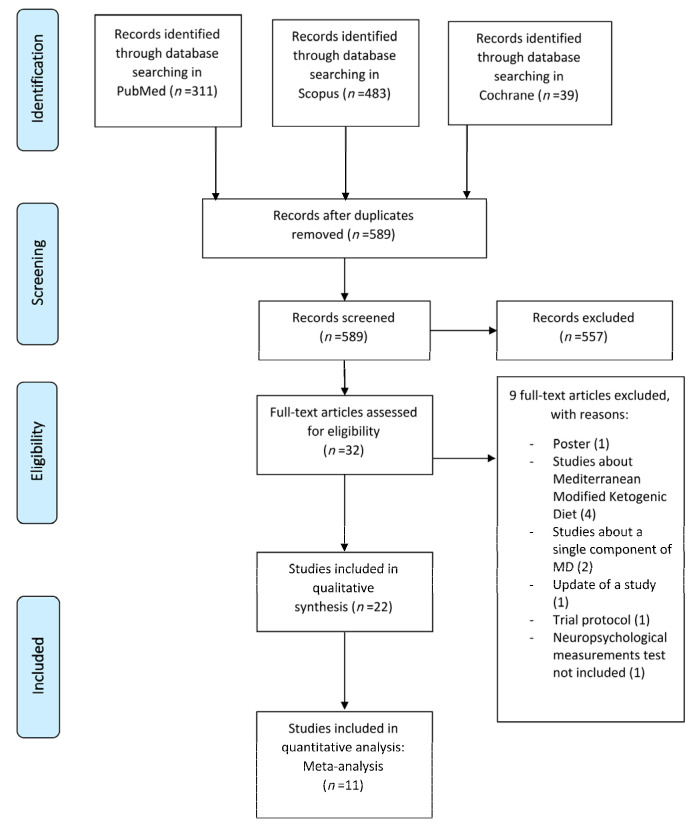
Flowchart of identification and selection of studies.

After excluding duplicate results, a total of 589 articles were selected for analyze its tittles and abstracts, generating a selection of 32 potential eligible studies proposed for complete reading. Out of the 32 articles assessed for eligibility, 22 studies met eligibility criteria for the systematic review and qualitative synthesis. Finally, 11 studies were included for the meta-analysis. The study flowchart is shown in Figure 1, and characteristics of these studies are summarized in Table 1.

### 3.1. Qualitative Synthesis

#### 3.1.1. Participants Demographic and Clinical Characteristics

Most studies included participants of both genders, except two [31,32]. Mean age of the participants in most studies was over 60 years old, except five of them [1,3,7,17,35] in which younger participants were included.

According to nationality, fourteen studies [1,3,7,8,10,13,17,20,27,33,35,36,38] included American participants, four studies [9,15,31,32] included Australian participants, one study [34] shows results of Swedish individuals, one study [11] is based on Brazilian results, other one the participants were German [30] and only one study was performed in France [37]. 

In most studies, participants were persons without any cognitive decline or dementia diagnosis at baseline, except one of them that included individuals with subjective memory impairment [33], three of them [10,11,16] that included MCI persons, and three studies [11,16,17] that included AD patients at the beginning of the study. The incident cases were usually diagnosed from NINCDS-ADRDA (National Institute of Neurological and Communicative Disorders and Stroke-Alzheimer’s Disease and Related Disorders Association) for AD and standard criteria for MCI [39]. 

#### 3.1.2. Study Design and Mediterranean Diet Adherence

Eight longitudinal studies [1,13,14,16,27,34,37,38], eleven cross-sectional articles [3,8,10,11,12,17,30,31,32,33,35] and one case-control study nested in a cohort [17] were included. Also, two studies [7,36] that analyzed its main associations cross-sectional and longitudinally were reviewed.

Evidence shows that dietary scores are useful tools to evaluate the degree of adherence to MD [40,41]. In order to quantify adherence to MD, food frequency questionnaires (FFQ) is currently the most frequent method used to assess food intake in large population-based studies and based on them, MedDiet scores (MDs) can be calculated, in which greater scores indicate higher MD adherence. In our revision to quantify the adherence to MD, most studies, administered a food frequency questionnaire (FFQ) to evaluate participant’s dietary intake.

#### 3.1.3. Effects of Mediterranean Diet Adherence on Neuropsychological Tests and Cognitive Function

In this systematic review, the neuropsychological tests used in the studies, focus on the evaluation of the different cognitive domains that mainly include immediate and delayed memory, executive function, attention, verbal fluency, information processing speed and global cognition. Some of them also include behavioral assessment, through depression or anxiety scales. The specific neuropsychological tests used in each study are reflected in Table 1.

Among the studies included, seven of them [1,10,11,16,20,30,37] analyzed associations between MD adherence and neuropsychological tests (NPT). Significant results were found in four of them. One study [10] showed that higher MD adherence was related with better learning and memory performance in non-demented subjects. Other study showed that higher MD adherence was related with better memory [30]. Another one [11] demonstrated association between higher adherence MD and higher Mini-Mental State Examination (MMSE) scores in Cognitively Normal (CN). In the fourth one [16], significant correlation was demonstrated between MD score and changes in MMSE in CN subjects.

#### 3.1.4. Effects of Mediterranean Diet Adherence on Magnetic Resonance Imaging Volumetry

Of the seven studies [1,3,7,10,20,30,35] that analyzed effects of MD on Magnetic Resonance Imaging (MRI) volumetry, four found associations between adherence to MD and cortical thickness [3,10,20,35]. Participants of these studies were all CN. 

#### 3.1.5. Effects of Mediterranean Diet Adherence on Glucose Metabolism in Brain

Three studies that analyzed effects of MD on glucose metabolism in brain were included in the present review, using all of them Fluoro-Deoxy-Glucose (FDG)-Positron Emission Tomography (PET) [1,7,35] as a neuroimaging technique and including only CN participants. These three studies found significant relations between lower adherence to MD and lower glucose metabolism at baseline [7,35] and higher rates of decline of glucose metabolism longitudinally analyzed [1,7]. 

#### 3.1.6. Effects of Mediterranean Diet Adherence on Brain Alzheimer’s Disease β-amyloid and Tau Tangles Deposition

Eight studies that used neuroimaging techniques to study AD biomarkers deposition in brain were reviewed. Five articles used Pittsburgh B compound (PiB)-PET to analyze βA plaques [1,7,8,14,35] in CN participants. Among PiB-PET articles, one did not find significant association [1]; two of them showed significant relation between higher MD adherence and lower PiB-PET deposition [8,14]; and other two provided significant relation between lower MD adherence and higher PiB-PET deposition [7,35]. Two articles used Fluoro 18 Florbetaben (F18F)-PET to study βA deposition in brain, but neither found significant associations [31,32]. One study used 2-(1-(6-[(2-[F-18]fluoroethyl)(methyl)amino]-2naphthyl)ethylidene)malononitrile (FDDNP)-PET, which is a technique that allows analysis of βA plaques and Tau tangles deposition in brain, showing that higher consume of MD is related with lower FDDNP-PET binding, both in MCI and subjective memory impairment participants [33]. 

On the other hand, only one study included the measurements of Aβ42 / 40 ratio, pTau181 in cerebrospinal fluid, finding that a greater adherence to the Mediterranean diet was related to a decrease in amyloid and tau in cerebrospinal fluid [30].

#### 3.1.7. Effects of Mediterranean Diet Adherence on Alzheimer’s Disease Risk, Incidence, or Progression from Mild Cognitive Impairment

Eight studies that analyzed risk of AD, its incidence or progression from MCI in relation to MD adherence were included. Five articles [8,17,27,36,38] provided significant associations, showing that higher MD adherence was related with lower risk of AD incidence in non-demented persons. One case-control study nested in a cohort showed that higher adherence to MD was related with lower risk of AD [9]. Another study investigated adherence to MD in CN, MCI and AD participants, and showed that persons with MCI or AD had significant lower adherence to MD [16]. Only one study did not find association between MD adherence and AD incidence, although it found potentially association between higher MD adherence and lower risk of developing all-type cognitive impairment [34].

#### 3.1.8. Vascular Risk and Lifestyle Variables Included in the Studies

Six studies included vascular risk and lifestyle results. One study showed that higher insulin sensitivity relates to greater cortical thickness [3], and another one found correlation between higher body mass index (BMI) and higher FDDNP-PET binding in MCI subjects [28]. The association between high MD adherence and lower levels of high-sensitivity C-reactive protein (CRP) was showed in the study by Gu et al. [36]. In another study [1], higher plasma homocysteine levels were significantly associated with faster decline in cognition. Furthermore, effects of exercise on AD were analyzed in four studies, in which higher physical activity was significantly related with lower FDDNP-PET binding in MCI subjects [33], and lower AD risk in non-demented participants [17]. One study found significant relationship between lower physical activity and higher βA deposition, and lower measures in FDG-PET and MRI volumetry [35]. Other lifestyle variable evaluated was intellectual enrichment, which was related with better cognitive performance in NPT in CN participants [3].

### 3.2. Quantitative Synthesis

#### 3.2.1. Risk of Bias

The overall risk of bias assessment of cross-sectional and longitudinal studies is shown in Appendix B (Table A1). The overall NOS score of cross-sectional studies was 6 indicating a moderate risk of bias. Concerning longitudinal studies (cohort and nested case-control studies), we observed a low risk of bias with NOS scores ranged from 7 to 9 points.

#### 3.2.2. Meta-analysis: Mediterranean Diet and Risk of Mild Cognitive Impairment and Alzheimer’s Disease

**Figure 2:**
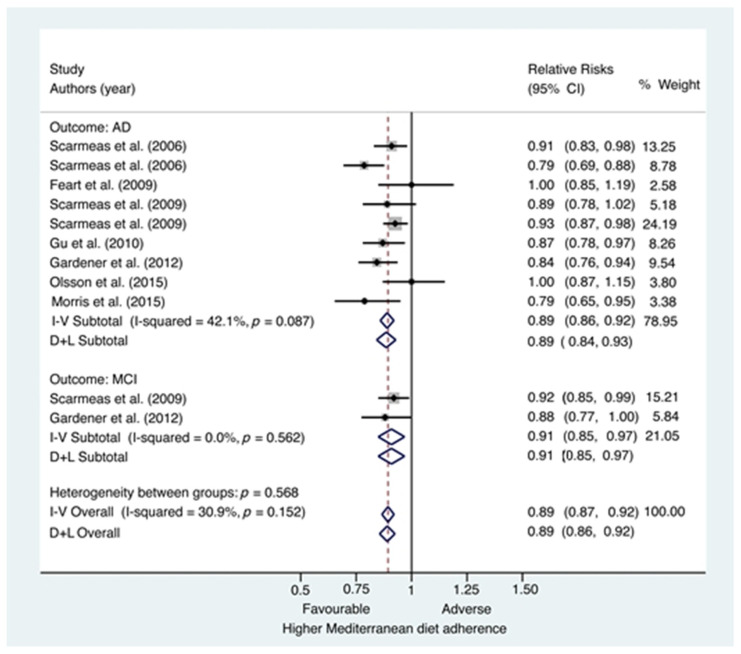
Forest plot of Mediterranean diet adherence and risk of Alzheimer’s disease or mild cognitive impairment. CI = confidence interval; AD = Alzheimer’s disease; I-V = Mantel-Haenszel method (fixed-effects model); D + L = DerSimonian and Laird method (random-effects model); MCI = Mild cognitive impairment.

Among studies examining AD (Figure 2), each one-point increase in the MD scores was associated with an 11% reduced risk of developing AD (RR = 0.89; 95%CI, 0.84–0.93). Figure 2 also reveals little visual evidence of heterogeneity among these studies despite quantitative evidence of moderate heterogeneity (I^2^ = 42.1%; *p* = 0.087).

When restricting the analysis to incident MCI (Figure 2), a higher MD adherence was also associated with a lower risk of developing MCI (RR = 0.91; 95%CI, 0.85–0.97) for each one-point increase in the MD score. We did not find neither visually nor quantitative evidence of heterogeneity among these studies (I^2^ = 0%; *p* = 0.562).

The analysis of the whole studies showed that each one-point increase in the MD scores was associated with an 11% reduced risk of developing AD or MCI (RR = 0.89; 95%CI, 0.86–0.92). A moderate evidence of heterogeneity was considered when we analyzed the whole studies (I^2^ = 30.9%; *p* = 0.152).

**Figure 3:**
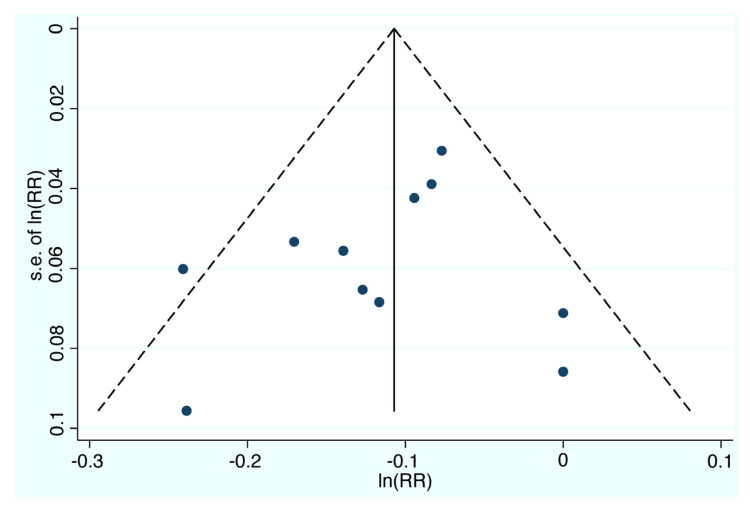
Funnel plot with pseudo 95% confidence limits. RR=relative risk; s.e. = standard error.

Visual inspection of the funnel plot (Figure 3) did not show asymmetry, an indication that significant publication bias was not likely. This was further confirmed by non-significantly both Egger’s and Begg’s tests (*p* = 0.935 and *p* = 0.876 respectively).

#### 3.2.3. Subgroup and Sensibility Analyses

**Figure A1:**
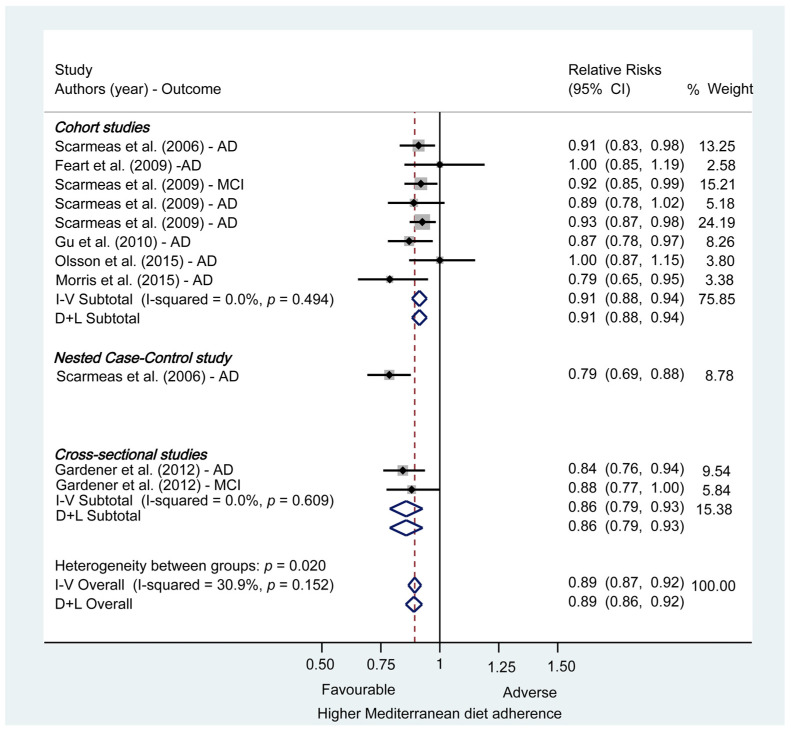
Mediterranean diet adherence and risk of Alzheimer’s disease or mild cognitive impairment: a subgroup analysis according to study design. CI = confidence interval; AD = Alzheimer’s disease; MCI = Mild cognitive impairment; I-V = Mantel-Haenszel method (fixed-effects model); D + L = DerSimonian and Laird method (random-effects model).

Subgroup and sensitivity analyses were conducted to examine the stability of the primary results (Figure 2). A subgroup analysis according to study design is shown in Appendix B (Figure A1). The associations between MD adherence and risk of AD or MCI were similar in subgroups. Among cohort studies, each one-point increase in the MD scores was associated with an 9% reduced risk of developing AD or MCI (RR = 0.91; 95%CI, 0.88–0.94). We did not find neither visually nor quantitative evidence of heterogeneity among these studies (I^2^ = 0%; *p* = 0.494). 

Sensitivity analysis that excluded three studies where ORs instead of HRs where presented [9,16] had little effect on the results (overall RR = 0.91; 95%CI, 0.88–0.94). When ORs were not transformed into RRs, the pooled RR estimated was practically equal to that obtained in our results (overall RR = 0.88; 95%CI, 0.85–0.92).

## 4. Discussion

The purpose of this systematic review and meta-analysis was to analyze through scientific evidence if adherence to MD is a beneficial factor to reduce the development of MCI and AD. To this end, 22 articles were qualitatively analyzed, and 11 were included in a meta-analysis. The pooled results showed that a higher adherence to MD significantly reduces the risk of developing MCI and AD.

Other previous systematic reviews and meta-analyzes have concluded that the highest Mediterranean diet score was inversely associated with cognitive decline [12,15,18]; Wu et al. in their meta-analysis, found a trend of a linear relationship of the Mediterranean diet score with the incident risk of cognitive disorders, but the association was not significant. In the analysis, additionally to MCI and AD, they also included other non AD type of dementia [18]; Furthermore Sigh et al. concluded that higher adherence to the MD was associated with a reduced risk of developing MCI and AD, and a reduced risk of progressing from MCI to AD. However, the overall number of studies were small (a total of five studies in the quantitative analysis) [12]. Fuerthermore, a recent study by, Charisis et al, demonstrated that individuals with highest adherence to MD had a 72% lower risk for development of dementia [42].

Up to date, prospective-cohort studies with longer follow-up has been performed. In order to consolidate greater evidence and to focus exclusively in AD type dementia and the previous stage of the disease (MCI), in the present review we first performed a qualitative analysis with a detail and comprehensive update from a clinical point of view of the studies in this field. In a second step we have deepened by carrying out a quantitative analysis to analyze the effects of the MD and the risk of developing MCI or AD. 

Among the 22 studies reviewed, a total of 19 articles showed significant associations supporting MD role in prevention of AD development. Concerning AD risk, incidence, or progression from MCI, seven studies provided significant evidence in relation to MD adherence as a protective factor [9,13,16,17,27,36,38]. Furthermore, three articles submitted evidence of association between MD adherence and better cognitive performance in NPT in CN participants [10,11,16] and one in MCI subjects [30]. In relation to AD preclinical biomarkers, it has been studied that AD preclinical biomarkers could appear 20–30 years upstream of clinical expression of AD [1]. In addition, it is suggested that changes in cortical thickness appear after decline in glucose metabolism and βA deposition in brain [3]. Five studies showed significant MD protective effects in MRI volumetry measures [3,10,20,30,35], three articles in glucose metabolism in brain [1,7,35], five studies in βA burden in brain [7,12,14,33,35], one study in Tau tangles deposition [33] and one related βA and Tau in cerebrospinal fluid [30].

These results, all of them obtained from studies whose participants were middle aged instead of elderly, suggest that pathophysiology of AD experiences an evolution from neurological metabolism dysfunction and pathological deposition of βA without cognitive impairment, to changes in brain structure, leading all these events to AD cognitive manifestations. 

There are non-modifiable factors that have been demonstrated to increase risk of AD and that have been included in some articles as covariates. In relation to them, nineteen articles included an APO-E4 genotyping [1,3,7,8,9,13,14,16,20,27,31,32,33,34,35,36,37,38], showing the study of Mosconi et al. that effects of greater cortical thickness in subjects with high adherence to MD were even greater in those that did not carry APO-E4 gene [20]. APO-E4 gene has been suggested to be a potential mediator of metabolic functions [31], so its presence could reduce protective effects of adherence to MD in people. With respect to age, most of the studies included elderly people, except five of them [1,3,7,20,35] which included CN middle aged participants. The last non-modifiable risk factor for AD that has been considered in articles is female gender, who is greater risk of developing AD and progressing from MCI to AD. A possible reason for that fact is that penetrance of APO-E4 is higher in women than in men [1,32]. Even though most of the studies included both female and male in their participants, two studies included only female [31,32] (although no significant associations were found) and one study included only male participants [34] (without significant relating MD to AD risk neither). 

The results of our meta-analysis should be interpreted in context of the limitations of available data. Modifiable risk factors are especially important influencing MD adherence protective effects, acting synergistically and additively to modify AD risk. Among them, cardiovascular risk factors, exercise, inflammation markers, BMI, years of education, smoking and comorbidities has been considered when analyzing main results in some studies. In addition, some of the studies, due to the difficulty to distinguish AD from vascular dementia, excluded from their participants those that suffered from cardiovascular diseases [1,3,7,11,20,24,34,35].

Furthermore, MD is the healthy dietary pattern object of study in this review. In most of the studies, FFQ were administered to participants to obtain information about their dietary habits and, after answering them, MDs were constructed therefrom. This procedure, although is based on the use of validated questionnaires and scores, have certain limitations. On one hand, cut-offs used for the construction of MDs depend on the average consume of each food product in the population studied, and that average is different between populations. Those dietary habits differences between populations makes difficult to compare MD adherences obtained from studies conducted in different countries. Furthermore, FFQ can underestimate consume of some food groups which might not be included or fully represented in their food lists. Only one study that fulfilled the inclusion criteria were conducted in a Mediterranean country, (France), where MD adherence is supposed to be greater [37].

Another limitation of these procedures used for quantifying MD adherence is that they rely on self-reported data. When AD subjects were included, their caregivers were who reported their food consume, but, despite this, cares are often elderly relatives of the patient and could also have some cognitive decline. It is also needed to consider that cognitive impairment patients could obtain MDs that do not accurately quantify their adherence to MD in the past. With this purpose, some studies evaluated stability of dietary habits in their participants [7,17,20,27,32,35,36,37,38].

There are underlying biological mechanism that could explain MD role in AD prevention. Even though they are still unknown, some studies propose four different pathways that could interplay in order to explain how MD is a protective factor for AD: MD could be neuroprotective through its metabolic effects, which is reflected in those studies that show significant associations between higher MD adherence and greater glucose metabolism in brain. Vascular pathways have also been studied as mediators of MD effects. MD has been demonstrated to reduce cardiovascular risk factors, which are themselves risk factors for AD [6]. On the other hand, MD has been proved to reduce oxidative stress due to antioxidative properties of many of its food components [34]. Finally, anti-inflammatory properties of MD could have an important role in its neuroprotective effects. 

Furthermore, other lifestyle habits, such as exercise, that have been proposed to be independent protective factors for AD, could interact with MD habits enhancing each other its beneficial effects, as it has been found in one study [17].

In summary, the main limitations found in the present review, are the heterogeneity and variability of the articles included in it, such as differences in samples size or studies designs, being most of them cross-sectional ones, which limit to infer causality; the use of diverse FFQ and procedures to estimate MD adherence; differences between outcomes measured and techniques used for these purposes; and differences between dietary habits in regions where studies were conducted. Furthermore, another potential limitation is that tools used for estimate MD adherence are based in self-reported data, which might lead to bias. 

To overcome these limitations and strengthen the evidence available so far, more longitudinal studies, with longer follow-up periods from earlier ages of life in preclinical stages; studies performed in Mediterranean countries (where is known that MD adherence is higher) and more studies to determine the different pathological pathways underlying MD effects on brain, are needed.

This study has some strengths worth highlighting: the rigorous search and selection strategy, the use of three blinded reviewers for article selection and two blinded reviewers for data extraction, and the assessment of risk and publication bias.

## 5. Conclusions

This meta-analysis shows that higher adherence to MD reduce the risk to develop MCI and AD. These results reinforce the need to make public health efforts and policies to promote the adoption of MD habits as beneficial measure for dementia. These dietary measures, in combination with other healthy lifestyle and cardiovascular risk factors interventions, should be applied in the earliest ages, as a brain protective intervention.

## Figures and Tables

**Table 1:** Characteristics of studies included in qualitative analysis of association of Mediterranean diet with Alzheimer’s disease and mild cognitive impairment.

First Author, Year and Country	Sample Size	Mean Age (Years)	Study Desing	Diet Measures: FFQ → Diet Score (Scale)/Pattern	Other Tests and Measures	Results
Ballarini et al., 2021 Germany [30]	N = 512	69.5	CS Groups: CN (N = 169) MCI (N = 81) SCD (N = 209) AD (N = 53)	EPIC-FFQ → MDs (1–9) -Low MDA ( 0–3) -Medium MDA (4–5) -High MDA (6–8)	-MRI volumetry -NPT (ADAS-COG; WMS; CERAD; SDMT; FNT; FCSRT; VF; BNT; TMT, CDT) -Cerebrospinal fluid (Aβ42/40 ratio, pTau181)	Higher MDA related to larger mediotemporal gray matter volume (*p* < 0.05), better memory (*p* = 0.038), and less amyloid (*p* = 0.008) and pTau181 pathology (*p* = 0.004)
Karstens et al., 2019 USA [10]	N = 82	68.8	CS Groups: ND (N = 82)	BFFQ 2005 → MDs (0–55) -Median split: -Low MDA (N = 39) -High MDA (N = 43)	-MRI volumetry -NPT (CVLT-II, TMT, WAIS-IV digit symbol coding, WAIS-IV letter number sequencing subtest, WTAR, MMSE, BDI, BAI) -MFSRP -BMI	High adherence to MD is related with better learning and memory in NPT (*p* = 0.007) and with larger dentate gyrus volumes compared with low MDA (*p* = 0.03). MDA is not related with information processing or executive functioning in NPT and neither with white matter hyperintensity.
Walters et al., 2018 USA [1]	N = 70	49	L for 3 years Groups: CN (N = 70)	HWSQFFQ → MDs (0–9) - Continuous variable	-MRI volumetry -FDG-PET -PiB-PET -NPT (WAIS digit symbol substitution, WAIS vocabulary, MMSE, paragraph recall, paired associates recall, object naming, design tests) - Vascular risk measures (BMI, blood pressure, plasma cholesterol/HDL ratio, plasma homocysteine QUICKI) -APO-E G -MLTAQ -Intellectual activity through life interview	Lower adherence to MD is related with faster decline in FDG-PET (*p* < 0.05). Adherence to MD is not related with NPT, PiB-PET or MRI measures. Exercise and intellectual activity are not related with changes in AD biomarkers or NPT.
Calil et al., 2018 Brazil [11]	N = 96	75.2	CS Groups: CN (N = 36) MCI (N = 30) AD (N = 30)	FFQ → MDs (0–55) -Tertiles: -Low MDA -Middle MDA -High MDA	-NPT (MMSE, BCSB, VF, CDT, GDS) -BMI	Higher adherence to MD is related with higher MMSE and BCSB Learning scores in CN group (*p* < 0.05). No associations are found between other NPT outcomes and MDs. No associations are found between dietary patterns and NPT outcomes in MCI or AD participants.
Hill et al., 2018 Australia [31]	N = 115	70	CS Groups: Women of the Women’s Health Ageing Project (N = 115)	DQESv2 → -High fat pattern (N = 24) -MD pattern (N = 31) -Junk food pattern (N = 24) -Low fat pattern (N = 35)	-F18F-PET -NPT (CERAD) -BMI -APO-E G	Adherence to junk food pattern was related with higher F18F-PET measures (*p* = 0.03). Other dietary patterns are not related with F18-PET.
Rainey-Smith et al., 2018 Australia [14]	N = 77	71.5	L for 3 years Groups: CN (N = 77)	CCVFFQ → MDs (0–9) -Continuous variable	-PiB-PET -APO-E G -BMI -NPT (MMSE)	Higher MDs is related with lower PiB-PET measures (*p* = 0.007).
Vassilaki et al., 2018 USA [8]	N = 278	77.7	CS Groups: CN (N = 278)	MB1995RHHFFQ → MDs (0–9) -Continuous variable	-PiB-PET -BMI -NPT	Higher MDs (*p* = 0.012), vegetable consumption (*p* = 0.002), Vitamin A (*p* = 0.003) and β-carotene intakes (*p* = 0.005) and moderate alcohol consumption (*p* = 0.03) are related with lower PiB-PET measures.
Berti et al., 2018 USA [7]	N = 70	50	CS and L for 3 years Groups: CN (N = 70)	HFFQ → MDs (0–9) -Median split: -Low MDA (N = 36) -High MDA (N = 34)	-NPT (GDS, HDRS, MMSE, CDR, WAIS digit symbol substitution, paired associates recall, paragraph recall, design tests, object naming) -MRI volumetry -FDG-PET -PiB-PET -Vascular risk measures (BMI, blood pressure, plasma cholesterol, triglycerides, plasma homocysteine, fasting glucose, hip-to-waist ratio, QUICKI, fasting glucose)	Low MDA is related with lower FDG-PET measures and higher PiB-PET measures compared with high MDA at baseline (*p* < 0.001). Low MDA is related with greater FDG-PET declines and PiB-PET increases compared with high MDA longitudinally (*p* < 0.001). No relation is observed between MDA and MRI volumes.
Hill et al., 2018 Australia [32]	N = 111	69.7	CS Groups: Women of the Women’s Health Ageing Project (N = 111)	DQESv2→ MDs (0–18 ) -Continuous variable -Tertiles: -Low MDA (N = 56) -Middle MDA (N = 32) -High MDA (N= 23)	-NPT -MRI -F18F-PET -IPAQ -BMI -IPAQ-E -AACVRs	There is no significant relation between MDA and F18F-PET measures.
Mosconi et al., 2018 USA [3]	N = 116	50	CS Groups: CN (N = 116)	BFFQ y HFFQ → MDs -Continuous variable	-MRI volumetry -NPT (CDR, GDetS, HDRS, memory, WAIS digit symbol substitution, WAIS vocabulary) -Intellectual activity through life 25-item interview -Vascular risk measures (BMI, blood pressure, plasma cholesterol, plasma homocysteine, QUICKI) -Baecke and Minnesota leisure time physical activity questionnaires	Higher MDA and higher insulin sensitivity are both significant related with higher MRI volumetry measures (*p* < 0.08). No other lifestyle and vascular risk variables are significant related with MRI volumetry measures. Higher MRI volumes are significant related with better cognitive performance. Intellectual enrichment is related with better cognition (*p* < 0.01)
Merrill et al., 2016 USA [33]	N = 44	62.6	CS Groups: SMI (N = 24) MCI (N = 20)	5 points Likert scale of Mediterranean-type diet -Often adherence to MD -Rarely adherence to MD	-FDDNP-PET -BMI -IPAQ-E -NPT (MMSE, HRSD, HRSA) -MRI	MCI group with above normal BMI have higher FDDNP-PET binding than MCI group with normal BMI (*p* = 0.02). Higher physical activity is related with lower FDDNP-PET binding in MCI group (*p* = 0.04) but not in SMI group. Higher consume of MD is related with lower FDDNP-PET binding in both groups (*p* = 0.04)
Morris et al., 2015 USA [27]	N = 923	58–98 (Range)	L for 4.5 years Groups: ND (N = 923)	HFFQ → MDs (0–55) -Continuous variables -Tertiles: -Low MDA -Middle MDA -High MDA	-NPT (CESDS) -APO-E G -BMI -Cognitively stimulating activities self-reported -Physical activity time spent self-reported	High adherence to MD have significant lower rates of AD incidence than low adherence to it (*p* for trend = 0.006).
Olsson et al., 2015 Sweeden [34]	N = 1038	71	L for 12 years Groups: Men CN (N = 1038)	Seven days food record prepared by Swedish National Food Administration→ modified MDs(0–8) -Continuous variables -Tertiles: -Low MDA -Middle MDA -High MDA	-NPT (MMSE) -APO-E G -Vascular risk measures (BMI, blood pressure, plasma cholesterol, HDL and LDL cholesterol, serum triglycerides, insulin sensitivity) -CRP levels	Higher MDA is potentially- not significantly- related with lower risk of developing all-type cognitive impairment (but not with AD or all-type dementia risk).
Matthews et al., 2014 USA [35]	N = 45	54	CS Groups: CN (N = 45)	HFFQ →MDs (0–9) -Median split: -Low MDA -High MDA	-MLTAQ -MRI volumetry -FDG-PET -PiB-PET -NPT (CDR, MMSE, HDRS, MHIS, WAIS vocabulary, WAIS digit symbol substitution, paired associated recall, paragraph recall, designs, object naming) -APO-E G -Vascular risk measures (BMI, HTWR, blood pressure, plasma cholesterol, HDL and LDL cholesterol, blood glucose, serum triglycerides, insulin sensitivity)	Lower physical activity is related with higher PiB-PET measures, lower FDG-PET measures and reduced MRI measures than higher physical activity (*p* < 0.001). Low MDA is related with higher PiB-PET measures, lower FDG-PET measures and reduced MRI measures than high adherence (*p* < 0.001). Significant interactions effects between physical activity and MDA are seen in FDG-PET measures (*p* = 0.003).
Mosconi et al., 2014 USA [20]	N = 52	54	CS Groups: CN (N = 52)	HFFQ → MDs (0–9) -Continuous variable -Median split: -Low MDA -High MDA	-MRI volumetry -NPT (CDR, MMSE, HDRS, MHIS, GDetS, WAIS vocabulary, WAIS digit symbol substitution, paired associated recall, paragraph recall, designs, object naming) -Vascular risk measures (BMI, HTWR, blood pressure, plasma cholesterol, HDL and LDL cholesterol, blood glucose, serum triglycerides, plasma homocysteine, insulin sensitivity) -APO-E G	High MDA is related with greater MRI measures in left hemisphere AD-vulnerable regions compared with low MDA (*p* = 0.026). MDA is not related with cognitive performance.
Gardener et al., 2012 Australia [16]	N = 970	71.72	CS Groups: CN (N = 723) MCI (N = 98) AD (N = 149)	CCVFFQ →MDs (0–9) -Continuous variable	-NPT (MMSE, LM II, D-KEFS Verbal Fluency, CVLT II Long Delay) -BMI -APO-E G	AD group has lower MDA than CN group (*p* < 0.001). MCI group has lower MDA than CN group (*p* < 0.05). MDs is related with changes in MMSE over 18 months period in CN group (*p* < 0.05).
Gu et al., 2010 USA [36]	N = 1219	76.7	CS and L for 4 years Groups: ND (N = 1219)	HFFQ → MDs (0–9) -Continuous variable -Tertiles: -Low MDA -Middle MDA -High MDA	-NPT (memory, language, processing speed and visual-spatial ability) -High sensitivity CRP plasma levels -Fasting insulin serum levels -Serum total adiponectin levels -APO-E G -BMI -Modified CIC	Higher MDA is related with lower levels of hsCRP (*p* = 0.003). Higher MDA is not related with levels of fasting insulin or total adiponectin. Higher MDA is related with lower risk of developing AD (*p* for trend = 0.04). Association between MDA and AD risk of incidence did not seem to be mediated by high sensitivity CRP, fasting insulin or total adiponectin levels.
Scarmeas et al., 2009 USA [17]	N = 1880	77.2	L for 14 years Groups: ND (N = 1880)	HFFQ →MDs (0–9) -Continuous variable -Tertiles: -Low MDA -Middle MDA -High MDA -Median split: -Low MDA -Middle MDA -High MDA	-NPT (memory, orientation, abstract reasoning, language, visual-spatial abilities, CDR) -GLTEQ -BMI -CIC -APO-E G	Middle MDA compared with low MDA reduces AD risk with HR= 0.98 (95% CI 0.72–1.33), while high MDA compared with low MDA reduces AD risk with HR = 0.6 (95% CI 0.42–0.87), (*p* for trend= 0.008). Some physical activity compared with no physical activity reduces AD risk with HR = 0.75 (95% CI 0.54–1.04), while much physical activity compared with no physical activity reduces AD risk with HR = 0.67 (95% CI 0.47–0.95) (*p* for trend = 0.03). Much physical activity and high MDA compared with no physical activity and low MDA reduces AD risk with HR = 0.65 (95% CI 0.44–0.96) (*p* for trend = 0.03).
Scarmeas et al., 2009 USA [13]	N = 1875	76.9	L for 10 years Groups: CN (N = 1393) MCI (N = 482)	HFFQ →MDs (0–9) -Continuous variable -Tertiles: -Low MDA -Middle MDA -High MDA	-NPT (memory, orientation, abstract reasoning, language, visual-spatial abilities, CDR) -BMI -APO-E G	Middle MDA compared with low MDA is borderline related with lower risk of developing MCI (*p* = 0.24). High MDA compared with low MDA is related with lower risk of developing MCI (*p* = 0.05). Middle MDA compared with low MDA is related with lower risk of developing AD from MCI (*p* = 0.01). High MDA compared with low MDA is related with lower risk of developing AD from MCI (*p* = 0.02).
Feart et al., 2009 France [37]	N = 1410	75.9	L for 5 years Groups: CN (N = 1410) AD (N = 66)	HFFQ →MDs (0–9) -Continuous variable -Tertiles: -Low MDA -Middle MDA -High MDA	-NPT (MMSE, IST, BVRT, FCSRT) -APO-E G	Higher MDA, was associated with slower MMSE cognitive decline but not with other cognitive tests this relationship was attenuated when adjusting for stroke. Higher MDA was not associated with risk for incident dementia.
Scarmeas et al., 2006 USA [9]	N = 1984	76.3	Nested Case-control Groups: ND (N = 1790) AD (N = 194)	HFFQ →MDs (0–9) -Continuous variable -Tertiles: -Low MDA -Middle MDA -High MDA	-NPT (memory, orientation, abstract reasoning, language, visual-spatial abilities, CDR) -APO-E G -BMI -Modified CIC -Vascular risk measures (BMI, plasma cholesterol, HDL and LDL cholesterol, blood glucose, serum triglycerides, plasma homocysteine, insulin sensitivity)	Higher MDA is related with lower risk of AD (*p* < 0.001). High and middle MDA are related with lower risk of AD compared with low MDA (*p* for trend < 0.001). Vascular variables do not change de magnitude of the association.
Scarmeas et al., 2006 USA [38]	N = 2258	77.2	L for 10 years Groups: ND (N = 2258)	HFFQ →MDs (0–9 pts.) -Continuous variable -Tertiles: -Low MDA -Middle MDA -High MDA	-NPT (memory, orientation, abstract reasoning, language, visual-spatial abilities, CDR) -APO-E G -BMI	Higher MDA is related with lower risk of AD incidence (*p* = 0.003). High and middle MDA are related with lower risk of AD incidence compared with low MDA (*p* for trend = 0.007).

ADAS-COG: Alzheimer’s Disease Assessment Scale; AACVRs: Australian Absolute Cardiovascular Risk Score; AD: Alzheimer Disease; APO-E G: Apolipoprotein E genotype; BAI: Beck Anxiety Inventory; BCSB: Brief Cognitive Screening Battery; BDI: Beck Depression Inventory; BFFQ: Block Food Frequency Questionnaire; BMI: Body Mass Index; BNT: Boston naming test; CCVFFQ: Cancer Council of Victoria Food Frequency Questionnaire; CDR: Clinical Dementia Rating; CDT: Clock Drawing Test; CERAD: Consortium to Establish a Registry for Alzheimer's Disease; CESDS: Center for Epidemiological Studies-Depression Scale; BVRT: Benton Visual Retention Test; CID: Charlson Index of Comorbidity; CN: Cognitively Normal; CRP: C-Reactive Protein; CS: cross-sectional; CVLT-II: Californian Verbal Learning Test Second Edition; D-KEFS: Delis-Kaplan Executive Function System; DQESv2: Dietary Questionnaire for Epidemiological Studies Version 2; FDDNP-PET: 2-(1-(6-[(2-[F-18]fluoroethyl)(methyl)amino]-2naphthyl)ethylidene)malononitrile Positron Emission Tomography; FCSRT Free and Cued Selective Reminding Test; FDG-PET: Fluoro-Deoxy-Glucose Positron Emission Tomography; EPIC: German adaptation of the semiquantitative European Prospective Investigation of Cancer. FFQ: Food Frequency Questionnaire; F18F-PET: Fluoro 18 Florbetaben Positron Emission Tomography; GDetS: Global Deterioration Scale; GDS: Geriatric Depression Scale; GLTEQ: Godin Leisure Time Exercise Questionnaire; GVT: Greek vocabulary Test; GVLT: Greek Verbal Learning test; HARS: Hamilton Anxiety Rating Scale; HDRS: Hamilton Depression Rating Scale; HFFQ: Harvard Food Frequency Questionnaire; hsCRP: high-sensitivity C reactive protein; HTWR: Hip to Waist Ratio; IST: Isaacs Set Test; IPAQ-E: International Physical Activity Questionnaire modified for older adults; JLO: Judgment of Line Orientation L: Longitudinal; LM II: Logical Memory II; MFSRP: modified Framingham Stroke Risk Profile; MB1995RHHFFQ: Modified Block 1995 Revision of the Health Habits Food Frequency Questionnaire; MCI: Mild Cognitive Impairment; MCG: medical college of Georgia complex figure test; MD: Mediterranean Diet; MDA: Mediterranean Diet Adherence; MDs: MedDiet score; MHIS: Modified Hachinski Ischemia Scale; MLTAQ: Minnesota Leisure Time Activity; MMSE: Mini-Mental State Examination; MRI: Magnetic Resonance Imaging; ND: non-demented; NPT: Neuropsychological tests; PiB-PET: Pittsburgh Compound B Positron Emission Tomography; pts.: points; QUICKI: Quantitative Insulin Sensitivity Check Index; SCD: Subjective Cognitive Decline; SDMT Symbol digit modalities test; SMI: Subjective Memory Impairment; TMT: Trail Making Test; VF: Verbal Fluency; WAIS-IV: Wechsler Adult Intelligence Scale IV; WTAR: Wechsler Test of Adult Reading; WMS: Wechsler Memory Scale.

## Appendix A

Electronic search strategy:

(1) Diet, Mediterranean [Mesh] OR Mediterranean Diet;
(2) Alzheimer Disease [Mesh] OR Mild Cognitive impairment OR Cognitive Dysfunction [Mesh] OR Cognitive Decline OR Alzheimer OR Cognitive Aging [Mesh];
(3) (1) and (2).

## Appendix B

**Table A1:** Risk of bias of the included studies using the Newcastle-Ottawa scale.

Authors (Year)	Type of Study	Selection	Comparability	Outcome/Exposure	Overall	Risk of Bias
***Ouctome: AD***						
Scarmeas et. al. (2006)	Nested Case-control	3	2	2	7	Low
Scarmeas et. al. (2006)	Prospective cohort	4	2	2	8	Low
Feart et. al. (2009)	Prospective cohort	4	2	2	8	Low
Scarmeas et. al. (2009)	Prospective cohort	3	2	3	8	Low
Scarmeas et. al. (2009)	Prospective cohort	3	1	3	7	Low
Gu et. al. (2010)	Prospective cohort	3	2	3	8	Low
Gardener et. al. (2012)	Cross-sectional	3	2	1	6	Moderate
Olsson et. al. (2015)	Prospective cohort	3	2	2	7	Low
Morris et. al. (2015)	Prospective cohort	3	2	3	8	Low
***Ouctome: MCI***						
Scarmeas et. al. (2009)	Prospective cohort	4	2	3	9	Low
Gardener et. al. (2012)	Cross-sectional	3	2	1	6	Moderate

AD: Alzheimer’s Disease; MCI: Mild Cognitive Impairment.
